# Tenecteplase versus standard medical treatment for minor stroke due to anterior-circulation medium-vessel occlusion: a multicenter observational study

**DOI:** 10.3389/fnins.2026.1931392

**Published:** 2026-08-31

**Authors:** He Jiang, Huijuan Zhang, Kai Qiu, Ying Liu, Yanguo Chen

**Affiliations:** 1 Department of Neurology, Dongtai People's Hospital, Yancheng, Jiangsu, China; 2 Department of Interventional Radiology, The First Affiliated Hospital with Nanjing Medical University, Nanjing, China; 3 Department of Neurology, The Affiliated Taizhou People's Hospital of Nanjing Medical University (Taizhou Clinical Medical School of Nanjing Medical University), Taizhou, China

**Keywords:** ischemic stroke, medium-vessel occlusion, minor, standard medical treatment, tenecteplase

## Abstract

**Purpose:**

To assess whether intravenous tenecteplase is associated with improved clinical outcomes compared with standard medical treatment (SMT) in patients with minor acute ischemic stroke (AIS) caused by medium-vessel occlusion (MeVO).

**Methods:**

We conducted a multicenter, retrospective observational study of consecutive patients with minor AIS, defined as a National Institutes of Health Stroke Scale score <6, and imaging-confirmed anterior-circulation MeVO. Patients were classified into the tenecteplase group or the SMT group (without intravenous thrombolysis) based on their initial treatment strategy. The primary outcome was excellent functional outcome at 90 days, defined as a modified Rankin Scale (mRS) score of 0–1. Secondary outcomes included functional independence at 90 days (mRS 0–2). Inverse probability of treatment weighting (IPTW)-adjusted analyses were performed to balance measured baseline characteristics between groups.

**Results:**

A total of 216 patients were included, of whom 64 received tenecteplase and 152 received SMT. After IPTW adjustment, tenecteplase was associated with a higher likelihood of excellent functional outcome at 90 days compared with SMT (70.3% vs. 53.3%; adjusted odds ratio [aOR], 2.07; 95% confidence interval [CI], 1.11–3.87). Tenecteplase was also associated with a higher rate of functional independence at 90 days (mRS 0–2: 81.3% vs. 64.5%; aOR, 2.39; 95% CI, 1.18–4.87). No statistically significant differences were observed in hemorrhagic transformation (5.2% vs. 7.0%) or symptomatic intracranial hemorrhage (3.4% vs. 4.3%).

**Conclusion:**

Among patients with minor AIS caused by anterior-circulation MeVO, tenecteplase was associated with better functional outcomes, without an apparent increase in hemorrhagic complications.

## Introduction

Patients with minor acute ischemic stroke (AIS) and persistent anterior-circulation medium-vessel occlusion (MeVO) present a frequent but challenging therapeutic dilemma (Prabhakaran et al., 2026). Although neurological deficits are mild, a low National Institutes of Health Stroke Scale (NIHSS) score does not necessarily indicate a benign vascular lesion or a low risk of subsequent disability. MeVO may involve eloquent cortical or subcortical territories, and preserved collateral flow may initially attenuate clinical severity while leaving tissue at risk (Nogueira et al., 2024). Consequently, these patients may remain at risk for infarct progression, early neurological deterioration (END), and poor functional outcomes (McDonough et al., 2021; Lakhani et al., 2026).

However, the optimal treatment strategy for this population remains uncertain. Standard medical treatment (SMT), including antiplatelet or anticoagulant therapy when clinically indicated, is generally considered to carry a lower risk of hemorrhagic transformation (HT), but it does not actively promote reperfusion (Ospel et al., 2020). Although endovascular thrombectomy (EVT) for MeVO has attracted increasing interest, its benefit remains uncertain in patients with minor deficits because the smaller and more distal target vessels create a narrower procedural safety margin. Recent randomized trials have not established routine EVT as the default strategy for unselected medium or distal vessel occlusion (Psychogios et al., 2025; Goyal et al., 2025; Clarençon et al., 2024). Therefore, an alternative approach that can promote reperfusion while avoiding immediate mechanical instrumentation remains clinically relevant. Prior randomized trials in broadly defined minor stroke populations have not supported routine intravenous thrombolysis (IVT), and evidence specifically addressing imaging-confirmed anterior-circulation MeVO remains limited (Khatri et al., 2018; Chen et al., 2023).

Tenecteplase, a genetically modified thrombolytic with greater fibrin specificity, longer half-life, and single-bolus administration, is a practical alternative to alteplase (Campbell et al., 2018). Systematic reviews and randomized studies suggest it may enhance early reperfusion and improve short-term neurological outcomes without substantially increasing HT risk in AIS (Rodriguez et al., 2024). However, whether these potential benefits extend to patients with minor AIS and anterior-circulation MeVO remains unclear. This multicenter retrospective observational study was undertaken to evaluate the effectiveness and safety of tenecteplase compared with SMT in this population.

## Methods

### Study design and patient selection

This multicenter retrospective observational study included consecutive patients treated at three stroke centers between January 2022 and December 2025. The study was conducted in accordance with the Declaration of Helsinki and reported in accordance with the Strengthening the Reporting of Observational Studies in Epidemiology guidelines. Patients were eligible for inclusion if they were aged 18 years or older, had a clinical diagnosis of AIS within 24 h of symptom onset, presented with minor neurological deficits, defined as a National Institutes of Health Stroke Scale (NIHSS) score <6, and had imaging-confirmed anterior-circulation MeVO. Anterior-circulation MeVO was prespecified as occlusion of the M2 or M3 segment of the middle cerebral artery (MCA) or the A2 or A3 segment of the anterior cerebral artery (ACA). Patients were excluded if they had baseline intracranial hemorrhage, posterior circulation occlusion, proximal large-vessel occlusion (LVO) involving the intracranial internal carotid artery or M1 segment of the middle cerebral artery, or isolated distal occlusion outside the prespecified MeVO definition. Patients were also excluded if they had a pre-stroke modified Rankin Scale (mRS) score >1, received alteplase or another thrombolytic agent other than tenecteplase, underwent immediate EVT as the initial treatment strategy, or had missing key baseline variables or unavailable 90-day follow-up data.

### Imaging assessment

All patients underwent a standardized one-stop multimodal computed tomography protocol at baseline, including noncontrast CT, CT angiography (CTA), and CT perfusion, before treatment decision-making. Early ischemic changes were assessed using the Alberta Stroke Program Early CT Score (ASPECTS) on baseline noncontrast CT. Perfusion imaging was processed using automated software, with ischemic core volume defined as relative cerebral blood flow <30% and hypoperfusion volume defined as Tmax >6 s. In patients with neurological deterioration, emergent noncontrast CT was performed immediately to distinguish hemorrhagic complications from suspected ischemic progression. Follow-up vascular imaging, including intracranial CTA or magnetic resonance angiography, was performed 48–72 h after stroke onset. Target-vessel recanalization was assessed by comparing follow-up vascular imaging with baseline imaging and was defined as restoration of antegrade flow or disappearance of the target occlusion in the initially occluded artery (Kharitonova et al., 2013). All imaging variables were assessed by experienced stroke neurologists or neuroradiologists who were blinded to clinical outcomes. Disagreements were resolved by consensus.

### Treatment groups

Patients were classified into the tenecteplase group or the SMT group according to the initial acute-phase treatment strategy. The tenecteplase group comprised patients who received intravenous tenecteplase as the initial pharmacological reperfusion therapy. Eligibility for IVT was determined according to local acute stroke protocols and contemporary guideline- or consensus-based criteria during the study period. In general, tenecteplase was preferentially considered in patients with a shorter treatment time window, no contraindications to thrombolysis, potentially disabling symptoms, no extensive early ischemic changes on baseline imaging, and a relatively proximal MeVO location. For patients with unknown onset, wake-up stroke, or presentation beyond the conventional 4.5-h window, treatment decisions were based on multimodal imaging findings and the overall clinical risk–benefit assessment. Tenecteplase was administered as a single intravenous bolus at 0.25 mg/kg, with a maximum dose of 25 mg. The SMT group comprised patients who did not receive IVT and were managed with SMT according to local practice and contemporary stroke guidelines. SMT included antiplatelet therapy, anticoagulation when clinically indicated, statin therapy, blood pressure and glucose management, and other supportive care. For patients who developed END, rescue EVT was considered when neurological worsening was judged to be attributable to ischemic progression and repeat imaging confirmed persistent target-vessel occlusion with potential benefit from reperfusion. The decision to initiate rescue EVT was made after discussion by the treating stroke team, taking into account clinical severity, imaging findings, and procedural risk.

### Outcomes

The primary outcome was excellent functional outcome at 90 days, defined as an mRS score of 0–1. Secondary outcomes included functional independence at 90 days, defined as an mRS score of 0–2; 90-day death; END within 24 h after stroke onset; and target-vessel recanalization at 48–72 h. The 90-day mRS score was obtained through outpatient visits, structured telephone interviews, or standardized follow-up assessments by trained neurologists who were unaware of the initial treatment strategy. END was assessed within 24 h after stroke onset and was defined as an increase of ≥4 points in the total NIHSS score or an increase of ≥2 points in any individual NIHSS item from baseline, after excluding neurological worsening attributable to intracranial hemorrhage or nonischemic systemic causes (Qiu et al., 2025). Target-vessel recanalization was defined as described in the Imaging Assessment section. Safety outcomes included HT and symptomatic intracranial hemorrhage (sICH), classified according to the Heidelberg Bleeding Classification (von Kummer et al., 2015).

### Statistical analysis

Continuous variables were summarized as mean ± standard deviation or median with interquartile range (IQR), as appropriate, and categorical variables were presented as frequencies and percentages. Baseline characteristics were compared between the tenecteplase and SMT groups using the Student’s *t* test or Mann–Whitney *U* test for continuous variables and the *χ*^2^ test or Fisher exact test for categorical variables. Because treatment allocation was not randomized, inverse probability of treatment weighting (IPTW) based on the propensity score was used to reduce baseline imbalances between groups. The propensity score was estimated using a multivariable logistic regression model incorporating prespecified baseline variables, including age, sex, pre-stroke mRS score, baseline NIHSS score, onset-to-door time, ASPECTS, occlusion site, ischemic core volume, and hypoperfusion volume. Stabilized inverse probability weights were calculated to estimate the average treatment effect (ATE) of tenecteplase versus SMT. Covariate balance before and after weighting was assessed using standardized mean differences (SMD), with an absolute value <0.10 indicating adequate balance. Clinical outcomes were analyzed in the IPTW-weighted cohort. Binary outcomes were evaluated using IPTW-weighted logistic regression models, with treatment group as the independent variable, and were reported as IPTW-adjusted odds ratios (aORs) with 95% confidence intervals (CIs). Patients who underwent rescue EVT after END were included in the analysis according to their initial treatment strategy. A sensitivity analysis was performed after excluding patients who underwent rescue EVT to evaluate the robustness of the primary findings. All statistical tests were two-sided, with a *p* value < 0.05 considered statistically significant. Statistical analyses were performed using R version 4.4.2 (R Foundation for Statistical Computing, Vienna, Austria).

## Results

### Study population and baseline characteristics

**Figure 1:**
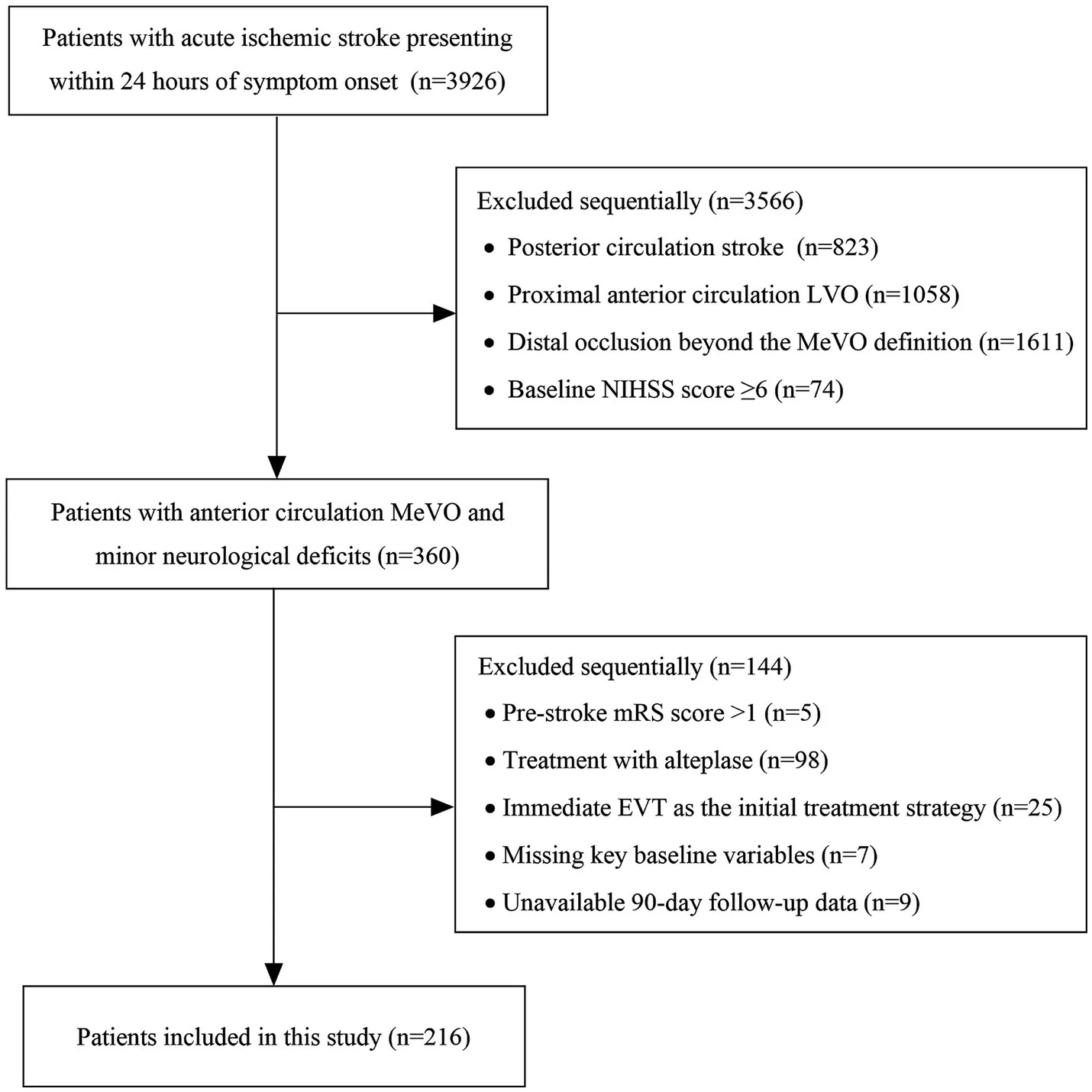
Flowchart showing the sequential selection of patients included in the study. LVO, large-vessel occlusion; MeVO, medium-vessel occlusion; NIHSS, National Institutes of Health Stroke Scale; mRS, modified Rankin Scale; EVT, endovascular thrombectomy.

The final study cohort comprised 216 patients, including 152 treated with SMT and 64 treated with tenecteplase (Figure 1). Overall, the mean age was 66.2 years, and 124 patients (57.4%) were men. The median baseline NIHSS score was 2 (IQR, 2–4), and the median baseline ASPECTS was 9 (IQR, 8–9). M2/M3 MCA occlusion was present in 149 patients (69.0%), and A2/A3 ACA occlusion was present in 67 patients (31.0%). Baseline characteristics before and after IPTW are shown in Table 1. Before IPTW, the SMT and tenecteplase groups were generally comparable, although patients treated with tenecteplase had a numerically higher NIHSS score and shorter onset-to-door time. After IPTW adjustment, measured baseline characteristics were well balanced between groups, with all absolute SMDs <0.10.

**Table 1:** Baseline characteristics before and after inverse probability of treatment weighting.

Variables	Before IPTW		After IPTW		
	Standard medical treatment (*n* = 152)	Tenecteplase (*n* = 64)	Standard medical treatment (*n* = 152)	Tenecteplase (*n* = 64)	SMD^#^
Age, mean (SD), years	66.5 ± 11.8	65.4 ± 9.2	66.2 ± 11.4	65.7 ± 10.8	0.045
Male, *n* (%)	89 (58.6)	35 (54.7)	88 (58.1)	36 (55.6)	0.050
Hypertension, *n* (%)	98 (64.5)	43 (67.2)	98 (64.8)	43 (66.5)	0.036
Atrial fibrillation, *n* (%)	25 (16.4)	8 (12.5)	24 (15.8)	9 (13.6)	0.062
Coronary heart disease, *n* (%)	19 (12.5)	9 (14.1)	19 (12.8)	9 (14.0)	0.035
Diabetes mellitus, *n* (%)	24 (15.8)	14 (21.9)	26 (17.1)	13 (20.3)	0.062
Previous stroke, *n* (%)	33 (21.7)	11 (17.2)	31 (20.7)	12 (18.6)	0.053
Prior antiplatelet, *n* (%)	40 (26.3)	18 (28.1)	41 (26.7)	18 (28.0)	0.029
Prior anticoagulant, *n* (%)	23 (15.1)	8 (12.5)	22 (14.7)	8 (13.0)	0.049
Pre-stroke mRS, median (IQR)	0 (0–0)	0 (0–0)	0 (0–0)	0 (0–0)	0.012
Baseline NIHSS, median (IQR)	2 (2–3)	3 (2–4)	2 (2–4)	3 (2–4)	0.073
Onset-to-door time, median (IQR), min	432 (293–572)	376 (247–493)	415 (282–555)	396 (261–532)	0.068
ASPECTS, median (IQR)	9 (8–9)	9 (8–9)	9 (8–9)	9 (8–9)	0.018
Occlusion site, *n* (%)					0.046
M2/M3 MCA	104 (68.4)	45 (70.3)	105 (68.8)	45 (70.9)	
A2/A3 ACA	48 (31.6)	19 (29.7)	47 (31.2)	19 (29.1)	
rCBF <30% volume, median (IQR), mL	3 (0–9)	4 (0–13)	3 (0–10)	4 (0–12)	0.065
Tmax >6 s volume, median (IQR), mL	32 (15–64)	39 (18–71)	34 (15–68)	38 (17–72)	0.072

^**#**^An absolute SMD <0.10 was considered indicative of adequate balance. IPTW, inverse probability of treatment weighting; SMD, standardized mean difference; SD, standard deviation; mRS, modified Rankin Scale; IQR, interquartile range; NIHSS, National Institutes of Health Stroke Scale; ASPECTS, Alberta Stroke Program Early CT Score; MCA, middle cerebral artery; ACA, anterior cerebral artery; rCBF, relative cerebral blood flow; Tmax, time to maximum of the residue function.

### Clinical outcomes

**Figure 2:**
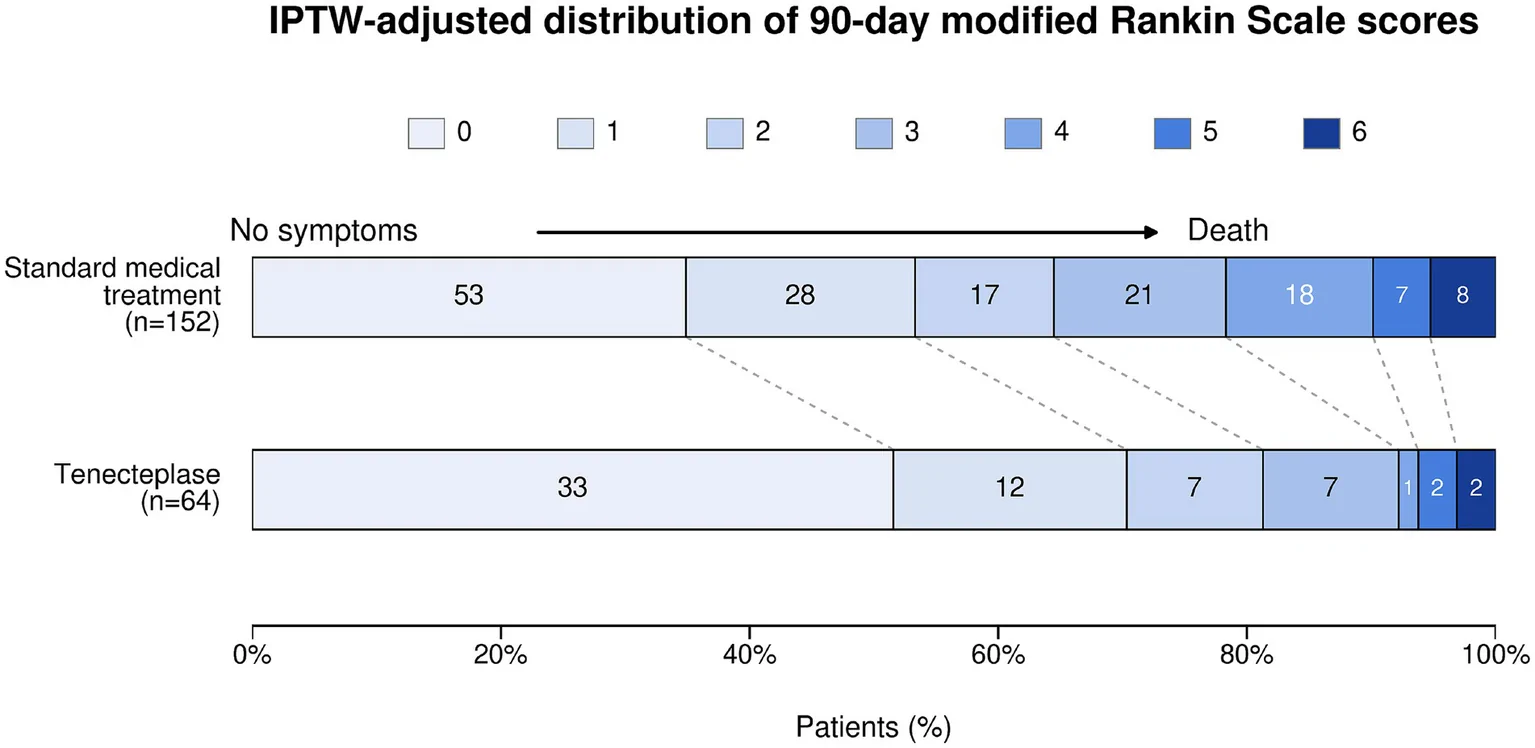
IPTW-adjusted distribution of 90-day modified Rankin Scale scores. Values represent IPTW-weighted counts rounded to the nearest integer. IPTW, inverse probability of treatment weighting.

**Figure 3:**
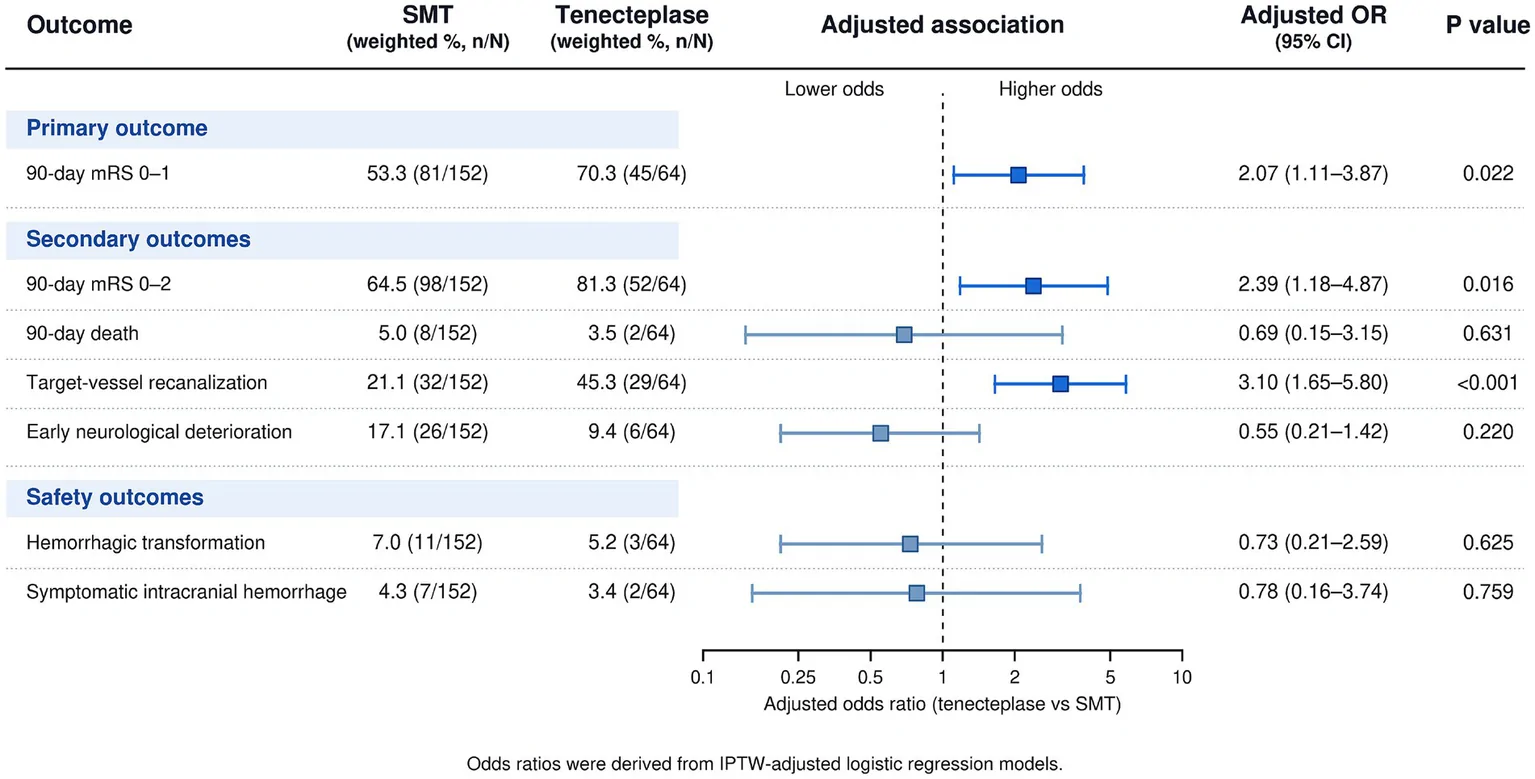
Adjusted treatment effects on clinical outcomes. Forest plot showing IPTW-adjusted treatment effects of tenecteplase versus standard medical treatment for efficacy and safety outcomes. For favorable efficacy outcomes, an odds ratio greater than 1 favors tenecteplase; for safety and adverse outcomes, an odds ratio less than 1 favors tenecteplase. SMT, standard medical treatment; IPTW, inverse probability of treatment weighting; OR, odds ratio; CI, confidence interval; mRS, modified Rankin Scale.

Clinical outcomes according to initial treatment strategy are presented in Table 2. The distribution of 90-day mRS scores is shown in Figure 2. After IPTW adjustment, tenecteplase was associated with a higher likelihood of excellent functional outcome at 90 days compared with SMT (70.3% vs. 53.3%; aOR, 2.07; 95% CI, 1.11–3.87; *p* = 0.022) (Figure 3). Tenecteplase was also associated with a higher likelihood of functional independence at 90 days (81.3% vs. 64.5%; aOR, 2.39; 95% CI, 1.18–4.87; *p* = 0.016). Ninety-day death occurred in 3.5% of patients in the tenecteplase group and 5.0% in the SMT group (aOR, 0.69; 95% CI, 0.15–3.15; *p* = 0.631). Tenecteplase was associated with a higher rate of target-vessel recanalization (45.3% vs. 21.1%; aOR, 3.10; 95% CI, 1.65–5.80; *p* < 0.001). END occurred less frequently in the tenecteplase group than in the SMT group, although the difference was not statistically significant (9.4% vs. 17.1%; aOR, 0.55; 95% CI, 0.21–1.42; *p* = 0.220). HT and sICH did not differ significantly between groups. After IPTW adjustment, HT occurred in 5.2% of patients in the tenecteplase group and 7.0% in the SMT group (aOR, 0.73; 95% CI, 0.21–2.59; *p* = 0.625). sICH occurred in 3.4 and 4.3% of patients, respectively (aOR, 0.78; 95% CI, 0.16–3.74; *p* = 0.759).

**Table 2:** Outcomes and IPTW-adjusted treatment effects according to initial treatment strategy.

Outcome	Before IPTW		After IPTW			
	Standard medical treatment (*n* = 152)	Tenecteplase (*n* = 64)	Standard medical treatment (*n* = 152)	Tenecteplase (*n* = 64)	adjusted OR (95% CI)	*p* value
90-day mRS 0–1, *n* (%)	84 (55.3)	43 (67.2)	81 (53.3)	45 (70.3)	2.07 (1.11–3.87)	0.022
90-day mRS 0–2, *n* (%)	101 (66.4)	51 (79.7)	98 (64.5)	52 (81.3)	2.39 (1.18–4.87)	0.016
90-day mRS 6, *n* (%)	8 (5.3)	2 (3.1)	8 (5.0)	2 (3.5)	0.69 (0.15–3.15)	0.631
Vessel recanalization, *n* (%)	30 (19.7)	31 (48.4)	32 (21.1)	29 (45.3)	3.10 (1.65–5.80)	<0.001
Early neurological deterioration, *n* (%)	25 (16.4)	6 (9.4)	26 (17.1)	6 (9.4)	0.55 (0.21–1.42)	0.220
Hemorrhagic transformation, *n* (%)	11 (7.2)	3 (4.7)	11 (7.0)	3 (5.2)	0.73 (0.21–2.59)	0.625
sICH, *n* (%)	7 (4.6)	2 (3.1)	7 (4.3)	2 (3.4)	0.78 (0.16–3.74)	0.759

IPTW, inverse probability of treatment weighting; OR, odds ratio; CI, confidence interval; sICH, symptomatic intracranial hemorrhage; mRS, modified Rankin Scale.

### Sensitivity analysis

Rescue EVT after END was performed in 7 patients in the SMT group and 2 patients in the tenecteplase group. Among patients who underwent rescue EVT in the SMT group, HT occurred in 2 patients, 2 achieved excellent functional outcome at 90 days, 4 achieved functional independence at 90 days, and 1 died within 90 days. Among patients who underwent rescue EVT in the tenecteplase group, no HT occurred, 1 patient achieved excellent functional outcome at 90 days, 2 achieved functional independence at 90 days, and no patient died within 90 days. After excluding patients who underwent rescue EVT, 145 patients remained in the SMT group and 62 patients remained in the tenecteplase group. The direction and magnitude of treatment effects were consistent with the primary analysis (Table 3). Tenecteplase remained associated with a higher likelihood of excellent functional outcome at 90 days compared with SMT (67.7% vs. 56.6%; aOR, 2.04; 95% CI, 1.08–3.86; *p* = 0.029). Tenecteplase also remained associated with a higher likelihood of functional independence at 90 days (79.0% vs. 66.9%; aOR, 2.27; 95% CI, 1.11–4.64; *p* = 0.025).

**Table 3:** Sensitivity analysis after excluding patients who underwent rescue endovascular thrombectomy.

Outcome	Standard medical treatment (*n* = 145)	Tenecteplase (*n* = 62)	Adjusted OR (95% CI)	*p* value
90-day mRS 0–1, *n* (%)	82 (56.6)	42 (67.7)	2.04 (1.08–3.86)	0.029
90-day mRS 0–2, *n* (%)	97 (66.9)	49 (79.0)	2.27 (1.11–4.64)	0.025
90-day mRS 6, *n* (%)	7 (4.8)	2 (3.2)	0.79 (0.17–3.69)	0.760
Hemorrhagic transformation, *n* (%)	9 (6.2)	3 (4.8)	0.90 (0.24–3.29)	0.868
sICH, *n* (%)	6 (4.1)	2 (3.2)	0.92 (0.19–4.53)	0.915

OR, odds ratio; CI, confidence interval; sICH, symptomatic intracranial hemorrhage; mRS, modified Rankin Scale.

## Discussion

In this multicenter retrospective study of patients with minor AIS and anterior-circulation MeVO, tenecteplase was associated with a higher likelihood of excellent functional outcome and functional independence at 90 days compared with SMT after IPTW adjustment. Tenecteplase was also associated with a higher rate of target-vessel recanalization, whereas HT, sICH, and 90-day death were not significantly increased. In a sensitivity analysis excluding patients who underwent rescue EVT, the direction of the associations remained broadly consistent.

The management of MeVO remains unsettled. Although EVT has become the standard of care for proximal LVO, its role in more distal occlusions is less certain. Recent randomized trials have challenged the assumption that the benefit of thrombectomy can be directly extended to medium or distal vessel occlusions. In DISTAL, EVT plus SMT did not reduce disability or death compared with SMT alone in patients with medium or distal vessel occlusion (Psychogios et al., 2025). ESCAPE-MeVO similarly did not demonstrate improved functional outcomes with EVT and reported numerically or significantly higher safety concerns, including mortality and symptomatic intracranial hemorrhage in the EVT group (Goyal et al., 2025). DISCOUNT was also stopped early and did not support routine thrombectomy for distal vessel occlusion (Clarençon et al., 2024).

These negative EVT trials do not mean that reperfusion is unimportant in MeVO. Rather, they highlight the need to identify a treatment strategy with an acceptable balance between benefit and risk. MeVO differs from proximal LVO in several clinically relevant ways, including smaller clot burden, smaller vessel caliber, more distal and tortuous anatomy, smaller but often eloquent ischemic territories, and greater procedural complexity. For patients presenting with minor deficits, the threshold for an invasive procedure is particularly high, because any treatment-related complication may offset the expected benefit (McDonough et al., 2021). At the same time, conservative medical treatment alone may be insufficient in patients with persistent arterial occlusion, especially when the occluded branch supplies functionally important cortex or when perfusion imaging suggests threatened tissue (Nogueira et al., 2024).

In this context, IVT may represent a pragmatic intermediate strategy between conservative medical treatment and immediate EVT. Compared with EVT, pharmacological reperfusion avoids mechanical manipulation of smaller distal vessels and may be more acceptable in patients with initially mild symptoms. Compared with antiplatelet- or anticoagulation-based medical treatment alone, thrombolysis provides an active reperfusion mechanism. This distinction is important when interpreting recent MeVO trials: in contemporary practice, “SMT” often includes IVT in eligible patients, rather than conservative therapy alone (Psychogios et al., 2025; Goyal et al., 2025; Clarençon et al., 2024). Thus, the unresolved clinical question is not simply whether MeVO should be treated with EVT, but which patients may benefit from pharmacological reperfusion, mechanical reperfusion, combined strategies, or careful medical observation. Tenecteplase may be particularly relevant in this setting. Its single-bolus administration, greater fibrin specificity, and longer half-life make it a practical thrombolytic option in acute stroke (Khatri et al., 2018). In MeVO, where clot burden may be smaller than in proximal LVO, pharmacological recanalization may be biologically plausible. In the present cohort, tenecteplase was associated with a substantially higher rate of vessel recanalization than SMT. This finding is consistent with the hypothesis that improved reperfusion may contribute to better functional outcomes.

Our findings should also be considered alongside prior trials of thrombolysis in minor stroke. PRISMS and ARAMIS did not support routine alteplase use in broadly defined minor nondisabling stroke populations (Khatri et al., 2018; Chen et al., 2023). More recently, TEMPO-2 did not show clinical benefit of tenecteplase in minor stroke with intracranial occlusion or focal perfusion abnormality and raised safety concerns (Coutts et al., 2024). These studies caution against indiscriminate thrombolysis for all patients with minor stroke. However, minor stroke is heterogeneous. Patients without persistent occlusion, patients with lacunar infarction, patients with transient embolic occlusion, and patients with imaging-confirmed MeVO may differ substantially in natural history and treatment responsiveness (McCarthy et al., 2021). The present study focused on a narrower population: patients with anterior-circulation MeVO confirmed by vascular imaging, generally small ischemic cores, and no immediate EVT as the initial treatment strategy. This selected phenotype may be more likely to benefit from pharmacological reperfusion, although this possibility requires prospective confirmation. The safety findings are clinically important but should be interpreted cautiously. In our study, tenecteplase was not associated with statistically higher rates of HT, sICH, or death. This finding is reassuring, particularly because the tolerance for treatment-related complications is low in patients presenting with minor deficits. However, the number of hemorrhagic events was small, the confidence intervals were wide, and this study was not designed or powered to establish safety equivalence. Therefore, the absence of a statistically significant safety signal should be regarded as supportive rather than definitive.

This study has several limitations. First, its retrospective observational design precludes causal inference. Although IPTW was used to balance measured baseline characteristics, residual confounding may remain. Nevertheless, the use of a multicenter consecutive cohort, prespecified eligibility criteria, IPTW adjustment, and sensitivity analysis excluding rescue EVT patients supports the robustness and internal consistency of the findings. Second, the sample size was modest, particularly in the tenecteplase group, which limited the precision of estimates for uncommon events such as HT and sICH and precluded reliable subgroup analyses. Third, MeVO was analyzed as a composite anterior-circulation phenotype including M2/M3 MCA and A2/A3 ACA occlusions, which may differ in clinical presentation, natural history, and treatment response. Fourth, recanalization was assessed at 48–72 h rather than at a uniform early post-treatment time point, limiting evaluation of early reperfusion dynamics. Finally, the findings may not be generalizable to posterior-circulation MeVO, patients with large infarct cores, patients selected for immediate EVT, those with high hemorrhagic risk, or centers without rapid multimodal imaging and rescue EVT capability. Prospective randomized studies with standardized imaging workflows and predefined rescue-treatment criteria are needed to confirm these findings.

## Conclusion

In this multicenter retrospective observational study of patients with minor AIS and anterior-circulation MeVO, tenecteplase use was associated with a greater likelihood of excellent outcome compared with SMT. Tenecteplase was also associated with higher rates of target-vessel recanalization, without an apparent increase in HT, sICH, or 90-day death. These findings suggest that tenecteplase may be a reasonable reperfusion strategy for selected patients with minor anterior-circulation MeVO, but prospective randomized studies are needed to confirm its efficacy and safety in this population.

## Data Availability

The raw data supporting the conclusions of this article will be made available by the authors, without undue reservation.
